# The value of the FDG-GaTate and proliferation marker (ki-67) in the assessment of neuroendocrine tumours (NETs)

**DOI:** 10.1186/1470-7330-15-S1-P53

**Published:** 2015-10-02

**Authors:** AS Fathinul Fikri, JN Abdul, S Ramdave, R Syafik-Eid

**Affiliations:** 1 Centre for Diagnostic Nuclear Imaging, Serdang, Selangor, Malaysia; 2 PET Centre, Moorabbin Hospital, MonashHealth, Melbourne, Australia

## Aims

A combined tracer evaluation of Ga-68 DOTATATE (GaTate) and F-18 FDG (FDG) positron emission tomography (PET-CT) and the ki-67 marker have potential advantages over a single marker in determining the differentiation of NETs. This study is sought to evaluate their association and the potential role as predictive markers for the management impact.

## Methods

Twenty-one combined FDG-GaTate studies were performed in various NETs lineages. A retrospective blinded review was performed based on the grade of tumour differentiation of ki-67 (European NET Society-ENETS) and the correlated Krenning scales (Grade 1-4) of the FDG-GaTATE PET-CT images. Subsequent management impact (high and low) was determined by follow-up to assess metabolic response of the pre and post treatment GATATE-FDG PET-CT results.

## Results

Significant correlation were noted in the Ki-67 (mean: 6.16± 8.21 %) and the FDG SUVmax (mean: 5.72±5.24;g/dl p < 0.01) and inversely correlated with the Ga SUVmax (mean: 15.80 ±10.57g/dl; p< 0.05). Management impact in 12/21 patients was high (partial metabolic response or no recurrence) in 75% and low in 25% (progressive metabolic disease). The combined ki-67-GaTATE marker had independent predictive significance for management impact (likelihood ratio test for the whole model, p=0.008).

## Conclusion

Dual-tracer assessment of FDG-GaTate PET-CT provide a valuable information on the NETs’ cellular differentiation. Combination of ki-67-GaTATE may potentially be used as a reliable predictive marker for the NETs’ management impact.

